# Immunophenotype of a Rat Model of Duchenne's Disease and Demonstration of Improved Muscle Strength After Anti-CD45RC Antibody Treatment

**DOI:** 10.3389/fimmu.2019.02131

**Published:** 2019-09-09

**Authors:** Laure-Hélène Ouisse, Séverine Remy, Aude Lafoux, Thibaut Larcher, Laurent Tesson, Vanessa Chenouard, Carole Guillonneau, Lucas Brusselle, Nadège Vimond, Karl Rouger, Yann Péréon, Alexis Chenouard, Christèle Gras-Le Guen, Cécile Braudeau, Régis Josien, Corinne Huchet, Ignacio Anegon

**Affiliations:** ^1^Centre de Recherche en Transplantation et Immunologie UMR 1064, INSERM, Université de Nantes, Nantes, France; ^2^Institut de Transplantation Urologie Néphrologie (ITUN), CHU Nantes, Nantes, France; ^3^Transgenesis Rat ImmunoPhenomic Facility, CRTI UMR 1064, Nantes, France; ^4^THERASSAY CAPACITES, Université de Nantes, Nantes, France; ^5^INRA, UMR703 APEX, Oniris, Ecole Nationale Vétérinaire, Agro-alimentaire et de l'alimentation, Nantes, France; ^6^Reference Centre for Neuromuscular Diseases AOC, CHU Nantes, Nantes, France; ^7^Pediatric Intensive Care, Hôpital Mère Enfant, CHU Nantes, Nantes, France; ^8^Clinical Investigation Center 1413 INSERM 1043, CHU Nantes, Nantes, France; ^9^CIMNA, Laboratoire d'Immunologie, CHU Nantes, Nantes, France; ^10^Thérapie Génique Translationnelle des Maladies Génétiques, INSERM UMR 1089, Nantes, France

**Keywords:** Treg, tolerance, muscle injury, dystrophin, immunosuppression, knockout rats, TALEN, nucleases

## Abstract

Corticosteroids (CS) are standard therapy for the treatment of Duchenne's muscular dystrophy (DMD). Even though they decrease inflammation, they have limited efficacy and are associated with significant side effects. There is therefore the need for new protolerogenic treatments to replace CS. Dystrophin-deficient rats (*Dmd*^*mdx*^) closely resemble the pathological phenotype of DMD patients. We performed the first Immunophenotyping of *Dmd*^*mdx*^ rats and showed leukocyte infiltration in skeletal and cardiac muscles, which consisted mostly of macrophages and T cells including CD45RC^high^ T cells. Muscles of DMD patients also contain elevated CD45RC^high^ T cells. We treated *Dmd*^*mdx*^ rats with an anti-CD45RC MAb used in previous studies to deplete CD45RC^high^ T cells and induce immune tolerance in models of organ transplantation. Treatment of young *Dmd*^*mdx*^ rats with anti-CD45RC MAb corrected skeletal muscle strength and was associated with depletion of CD45RC^high^ T cells with no side effects. Treatment of young *Dmd*^*mdx*^ rats with prednisolone resulted in increase in skeletal muscle strength but also severe growth retardation. In conclusion, anti-CD45RC MAb treatment has potential in the treatment of DMD and might eventually result in reduction or elimination of CS use.

## Introduction

Duchenne muscular dystrophy (DMD) is the most common inherited muscle disease. It is caused by a mutation in the dystrophin gene with X-chromosomal recessive inheritance and affects 1 in 3,500 males (1). It has a severe prognosis; life expectancy ranges from the late teens to mid-30s. Muscle fibers show necrosis and regeneration/degeneration associated with chronic inflammation, with progressive replacement by connective and adipose tissue (1).

The *mdx* mouse, which carries a mutation in the *Dmd* gene, is a well-established mouse model of DMD. Nevertheless, muscle impairment is rather mild in *mdx* mice compared to DMD patients. For this reason, new models of *mdx* mice with more severe disease have been developed [e.g., D2/*mdx* model; (2)]; however, new animal models are still required (3).

*Dmd-*deficient (*Dmd*^*mdx*^) rats, which we have previously generated using TALENs (4), represent a useful small animal model for DMD pre-clinical research (5). Forelimb and hindlimb muscular strength and spontaneous activity are decreased in these rats, and skeletal and cardiac muscles show necrosis and regeneration of muscle fibers associated with progressive replacement by fibrotic and adipose tissue. The weak muscle strength and muscular lesions therefore closely mimic those observed in DMD patients.

To date, there is no cure for DMD. Gene and cell therapies may cure the disease in the future, but there remains a need for therapies that target associated pathologies such as immune responses and inflammation. Immune responses are involved in disease pathophysiology both in DMD patients and *mdx* mice (6). Standard therapy for DMD is treatment with corticosteroids (CS). CS have been shown to act partly through anti-inflammatory mechanisms and through inhibition of CD8^+^ T cells, improving muscle strength in a fraction of patients (6–8). Thus, CS have moderate efficacy. They are also associated with serious systemic side effects, including short stature, obesity, psychological symptoms, osteoporosis, diabetes, and hypertension (7). Furthermore, through their broad and non-specific anti-inflammatory effects, CS inhibit inflammatory mechanisms that promote muscle repair (6).

The presence of T effector cells against DMD has been described in patients before and after gene therapy (9–11). CD4^+^ T regulatory cells (Tregs) limit disease severity in *mdx* mice through tissue repair activity as well as inhibition of immune responses (6, 12, 13).

Thus, inhibition of immune responses and promotion of immune tolerance are potentially important adjuvants to the DMD therapeutic arsenal. These immunointerventions however, should simultaneously preserve immune responses that promote muscle regeneration and protection against pathogens and cancer cells. Knowledge of immune responses in DMD patients and animal models are thus important for the development of targeted immunointerventions associated with other treatments such as gene or cell therapy. Furthermore, immune responses may be an obstacle to gene and cell therapy as newly produced dystrophin may be recognized as immunogenic leading to destruction of the cells which express it (11). Transient immunosuppression is being used in ongoing clinical trials in order to prevent these immune responses. Thus, analyses of immune cells and immunotherapies in *Dmd*^*mdx*^ rats could result in important developments and new treatments for DMD patients.

We have previously reported CD4^+^ and CD8^+^ Tregs in rats and humans as a subset of CD45RC^low/−^ cells (14, 15). We have also recently showed that treatment with an anti-CD45RC monoclonal antibody (MAb) induced permanent allograft acceptance in a rat model and inhibition of graft vs. host disease (GVHD) in a humanized mouse model (15). Anti-CD45RC treatment only depleted T cells that were CD45RC^high^ (i.e., naïve T cells, precursors of Th1 cells, and effector memory T cells including TEMRA cells). In contrast, CD45RC^low/−^ T cells were not depleted, possibly due to low antigen density. CD8^+^ and CD4^+^ Tregs in both rats and humans are CD45RC^low/−^ and were thus spared. CD8^+^ and CD4^+^ Tregs specific for donor alloantigens protected against graft rejection. Importantly, immune responses against third party donors and exogenous antigens were preserved. Thus, anti-CD45RC antibody treatment does not result in broad immunosuppression but rather specific elimination of T cells with effector functions and preservation of Tregs followed by their activation and expansion (15).

We thus reasoned that treatment of *Dmd*^*mdx*^ rats with anti-CD45RC MAbs could be beneficial to reduce muscle destructive mechanisms. To the best of our knowledge, treatment with antibodies directed against other cell antigens (e.g., anti-CD3, -CD28, -CD127, or -CD137) that promote immune tolerance in transplantation, GVHD, or autoimmune diseases has not been reported in other animal models of DMD. Thus, we aimed to describe normal baseline immune parameters in *Dmd*^*mdx*^ rats and assess how treatment with anti-CD45RC MAb affected muscle strength.

We observed that the skeletal and cardiac muscle of *Dmd*^*mdx*^ rats showed a leukocyte infiltrate predominantly consisting of macrophages and to a lesser extent by T cells. M2 type macrophages increased over time. Treatment with an anti-CD45RC depleting MAb resulted in increased muscle strength associated with a decrease in T cells but not of macrophages. Prednisolone treatment also increased muscle strength and decreased CD45RC^high^ cells but suppressed growth of *Dmd*^*mdx*^ rats whereas anti-CD45RC did not. Elevated CD45RC^+^ cells are also present in the blood and muscles of DMD patients.

In summary, immune responses and inflammation are present in the *Dmd*^*mdx*^ rat muscles and anti-CD45RC MAb treatment resulted in amelioration of skeletal muscle strength. This is the first report showing that treatment with a MAb targeting specific T cell sub-populations results in amelioration of clinical parameters in a faithful animal model of DMD.

## Materials and Methods

### Animal Experiments and Ethical Approval

*Dmd*^*mdx*^ rats have been described previously (4). *Dmd*^*mdx*^ and wild-type littermates were housed in specific-pathogen-free conditions in a controlled environment (temperature 21 ± 1°C, 12-h light/dark cycle). All animal care procedures were approved by the Animal Experimentation Ethics Committee of the Pays de la Loire region, France, in accordance with the guidelines from the French National Research Council for the Care and Use of Laboratory Animals (Permit Numbers: CEEA-PdL-10792 and CEEA-PdL-8986). All efforts were made to minimize suffering.

Blood samples, taken as part of standard clinical practice, were obtained from two DMD patients at the Nantes University Hospital. Informed consent was acquired from patients and their parents. Control blood samples were obtained from the pediatric bio-collection (Ref: MESR DC-2011-1399) managed by the University Hospital of Nantes and approved by the local ethics committee. Controls consisted of children without immune-related pathologies admitted to the Nantes University Hospital. No child's legal representative objected to them taking part in this bio-collection. Tissue samples were obtained from the *Paravertebralis* muscle of four 12-year old patients (two DMD patients and two patients free of known muscular disease). Patients underwent surgical procedures at the Department of Pediatric Surgery of the Nantes University Hospital de Nantes (France). Written informed consent was obtained. All protocols were approved by the Clinical Research Department of the CHU (Nantes, France), in accordance with the rules of the French Regulatory Health Authorities (Permit numbers: MESR/DC-2010-1199). The biobank was regulated in compliance with the national guidelines regarding the use of human tissue for research (Permit numbers: CPP/29/10).

### Preparation of Muscle and Spleen Single-Cell Suspensions

Muscles from both hindlimbs of WT or *Dmd*^*mdx*^ rats were excised, trimmed of adipose tissue, rinsed with PBS, and weighed. Muscles were minced at room temperature, placed in gentleMACS C tubes (Miltenyi Biotec) with collagenase D (4 ml/g of muscle), and dissociated using the gentleMACS^TM^ dissociator (program “m_muscle_01”). Samples were then rotated for 30 min at 37°C. Undigested muscle was collected on a mesh strainer and re-digested with fresh collagenase for another 30 min. The resulting filtered cell suspensions from both runs were centrifuged and re-suspended in PBS-FCS 2%-1 mM EDTA, and then gently applied onto 15 ml Histopaque 1077 density gradient (Eurobio) and centrifuged at 1,000 x g for 30 min. Interface cells were collected, washed, re-suspended in PBS-FCS 2%-1 mM EDTA, and counted.

Spleens were harvested, perfused with collagenase D, minced, and incubated for 15 min at 37°C as previously described (16). Spleen fragments were suspended in PBS-FCS 2%-1 mM EDTA and then forced through a mesh filter. Mononuclear cells were recovered using a density gradient (Histopaque 1077, Eurobio). Interface cells were collected, washed, re-suspended in PBS-FCS-2%-1 mM EDTA, and counted.

### Staining of Rat Cells for Flow Cytometry Analysis

Cytofluorimetry analysis was performed as previously described (16). In brief, single-cell suspensions from muscle or spleen were stained with MAbs against the following antigens: CD45 as a pan leukocyte (clone OX-1), TCRαβ (clone R7/3), CD45RA on B cells (clone OX33), CD45R/B220 on B cells (clone His24), anti-granulocytes (RP-1 and His48), CD4 (clone w3/25), CD45RC (clone OX22 or clone OX32), CD25 (clone OX39), CD8 (clone OX8), CD172a/SIRPα (clone OX41), CD161 on NK and myeloid cells (clone 3.2.3), CD163 on macrophages (clone ED2), CD68 for macrophages (clone ED1), and with viability dye eFluor506 or eFluor450 from eBiosciences to assess cell viability. Analysis was performed on a BD FACS Verse with FACSuite Software version 1.0.6. Post-acquisition analysis was performed using FlowJo software. The CD45RC^−^ population was defined using an isotype control. The CD45RC^high^ population was defined using as reference the levels of CD45RC expression on B cells since they always express the highest levels of CD45RC. The CD45RC^low^ population corresponded to the intermediate population between the CD45RC^−^ and the CD45RC^high^ cells.

### Serum Creatinine Phosphokinase and Cytokine Levels

Blood was collected while the rats were under anesthesia. Serum was isolated and immediately frozen at −20°C. Total creatinine phosphokinase (CK) activity was assessed by the Biochemistry Department of the Nantes University Hospital.

Levels of IL-1β, IL-6, IL-10, and TNFα in the serum of *Dmd*^*mdx*^ and WT littermate rats were measured by multiplex assays (Luminex technology, R&D systems) in accordance with the manufacturer's instructions.

### Quantitative RT-PCR

Quantification of mRNA levels was performed as previously described (17). Briefly, total RNA was extracted from skeletal muscle mononuclear cells using a RNeasy Mini Kit (Qiagen) according to the manufacturer's instructions. Quantification and quality analysis were assessed on a Caliper LabChip GX II (PerkinElmer). RNA with a quality score between seven and 10 were retro-transcribed using oligo-dT and M-MLV reverse transcriptase (Life Technologies). Fast SybrGreen Master Mix 2x was used to perform qPCR on a ViiA 7 (Applied Biosystems) on duplicate cDNA samples for each target according to the manufacturer's instructions. qPCR reaction conditions were 20 s at 95°C followed by 40 cycles of 1 s at 95°C, 20 s at 60°C, and 20 s at target melting temperature minus 3°C, followed by a final melt curve analysis step. Changes in relative gene expression between samples and treatments were calculated using the 2^−ρρ*Ct*^ method and normalized to HPRT house-keeping gene. The reference population included pools of immune cells from WT animals aged 8 or 12 weeks. The primers used in this study are listed in Supplementary Table I.

### Immunohistological Analysis and Fibrosis Quantification

Immunohistochemistry was performed as previously described (4). Briefly, tissue samples of *Biceps femoris* and cardiac ventricular muscles were harvested at 8 and 12 weeks of age, snap frozen, and sectioned (8-μm) for immunofluorescence labeling. Sections were pre-fixed in acetone (100%) for CD3 labeling and acetone/methanol (30%/70% v/v) for CD68, CD163, and CD45RC labeling (10 min, room temperature), and incubated with 0.2% triton in PBS (10 min, room temperature). Sections were then blocked with 10% goat serum in PBS and incubated with the primary antibodies. Rabbit polyclonal antibody for CD3 (DakoCytomation, Glostrup, Denmark) and mouse monoclonal antibodies for rat CD68, CD163, and CD45RC were used at 1:50, 1:200, and 1:200, respectively (overnight, 4°C). After washing, goat anti-rabbit and goat anti-mouse antibodies coupled with Alexa 488 (Invitrogen, Carlsbad, CA) were used to reveal CD3 and CD68 primary antibody, respectively (1 h, room temperature). Sections were incubated with wheat germ agglutinin Alexa Fluor 555 conjugate for connective tissue labeling (Molecular Probes, Eugene, OR) diluted 1:700 in PBS (overnight, 4°C), and nuclei were then labeled with Draq5 (BioStatus Ltd, Shepshed, UK) diluted at 1:1,000 (10 min, room temperature). Immunofluorescence labeling was analyzed with a laser scanning confocal microscope (Zeiss, LSM880, Jena, Germany) and with software Zeiss Zen Black edition (Zen 2.3 SP1 FP1).

Biopsies of human muscle were obtained from DMD patients undergoing surgery for spinal deformities and from young individuals undergoing muscle biopsy for other diagnoses. Tissues were snap frozen, sectioned, and processed as described above for rat tissues using an anti-human CD45RC MAb (BD Biosciences).

### Treatment With Anti-CD45RC and Prednisolone

WT and *Dmd*^*mdx*^ rats received intraperitoneal injections of the anti-rat CD45RC MAb (clone OX22, mouse IgG1) or an isotype control MAb (clone 3G8, mouse IgG1) at a dose of 1.5 mg/kg every 3.5 days from age 2 to 12 weeks as previously described (15). Prednisolone was administered by daily intraperitoneal injections at a dose of 0.5 mg/kg [similar to the 1 mg/kg dose in *mdx* mice (18) and 0.75 mg/kg in DMD patients (19)], from age 2 to 12 weeks.

### Grip Test

A grip test was performed as previously described (4). The *in vivo* tests were performed in the same sequence for each rat, with equivalent rest time in between (20). Rats were placed with their forepaws or four paws on a grid and were gently pulled backward until they released their grip (4, 21). The peak force generated was measured by a grip meter (Bio-GT3, BIOSEB, France) attached to a force transducer. Five tests were performed sequentially with a short latency between each. The reduction in strength between the first and the last test represented the index of fatigue (4). Results are expressed in grams (g) and normalized to the body weight (g/g). The observer was blinded to the treatment of the animals.

### Statistical Analyses

The Mann–Whitney *t*-test was used to compare cell numbers in the muscle and spleen and cytokine levels in sera of WT vs. *Dmd*^*mdx*^ rats. The two-way ANOVA test was used to compare growth curves. Unpaired *t*-tests were used to compare CK in sera.

## Results

### Increased Mononuclear Leukocyte Infiltration in Skeletal Muscles of *Dmd^*mdx*^* Rats

**Figure 1:**
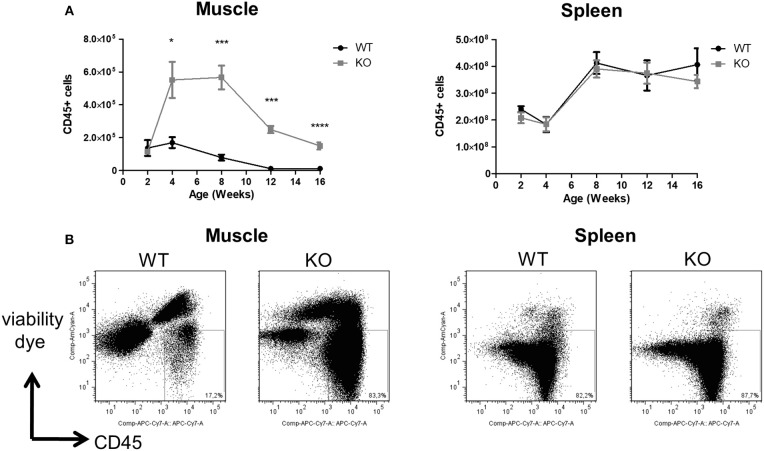
Numbers of leukocytes in the skeletal muscle and spleen of *Dmd*^*mdx*^ rats. Hind limb muscles and spleen were harvested from littermate wild-type (WT) or *Dmd*^*mdx*^ (KO) rats at the indicated time points. Muscle and spleen were digested with collagenase, mononuclear cells isolated using a density gradient, and analyzed by cytofluorimetry. **(A)** Number of viable CD45^+^ cells per gram of muscle (left panel) or whole spleen (right panel) at different time points. WT (*n* = 4, 5, 7, 7, 9), at 2, 4, 8, 12, and 16 weeks, respectively; *Dmd*^*mdx*^ (*n* = 3, 6, 10, 11, 16), at 2, 4, 8, 12, and 16 weeks, respectively, ^*^p < 0.05, ^***^p < 0.001 and ^****^p < 0.0001. **(B)** Representative dot-plot analysis of viable SSC vs. CD45^+^ mononuclear leukocytes from muscle (left panel) or spleen (right panel) from animals at 12 weeks of age.

The total number of CD45^+^ mononuclear leukocytes was similar in the skeletal muscle of littermate WT and *Dmd*^*mdx*^ rats at 2 weeks of age. A sharp increase was seen however at 4 weeks of age in *Dmd*^*mdx*^ rats which was maintained until week 8 but then decreased at weeks 12 and 14 to values that were still significantly higher than those observed in littermate WT rats (Figures 1A,B). Total leukocyte numbers in the spleen were comparable between WT and *Dmd*^*mdx*^ rats (Figure 1A).

### Macrophages and T Cells Are Elevated in Skeletal Muscle of *Dmd^*mdx*^* Rats

**Figure 2:**
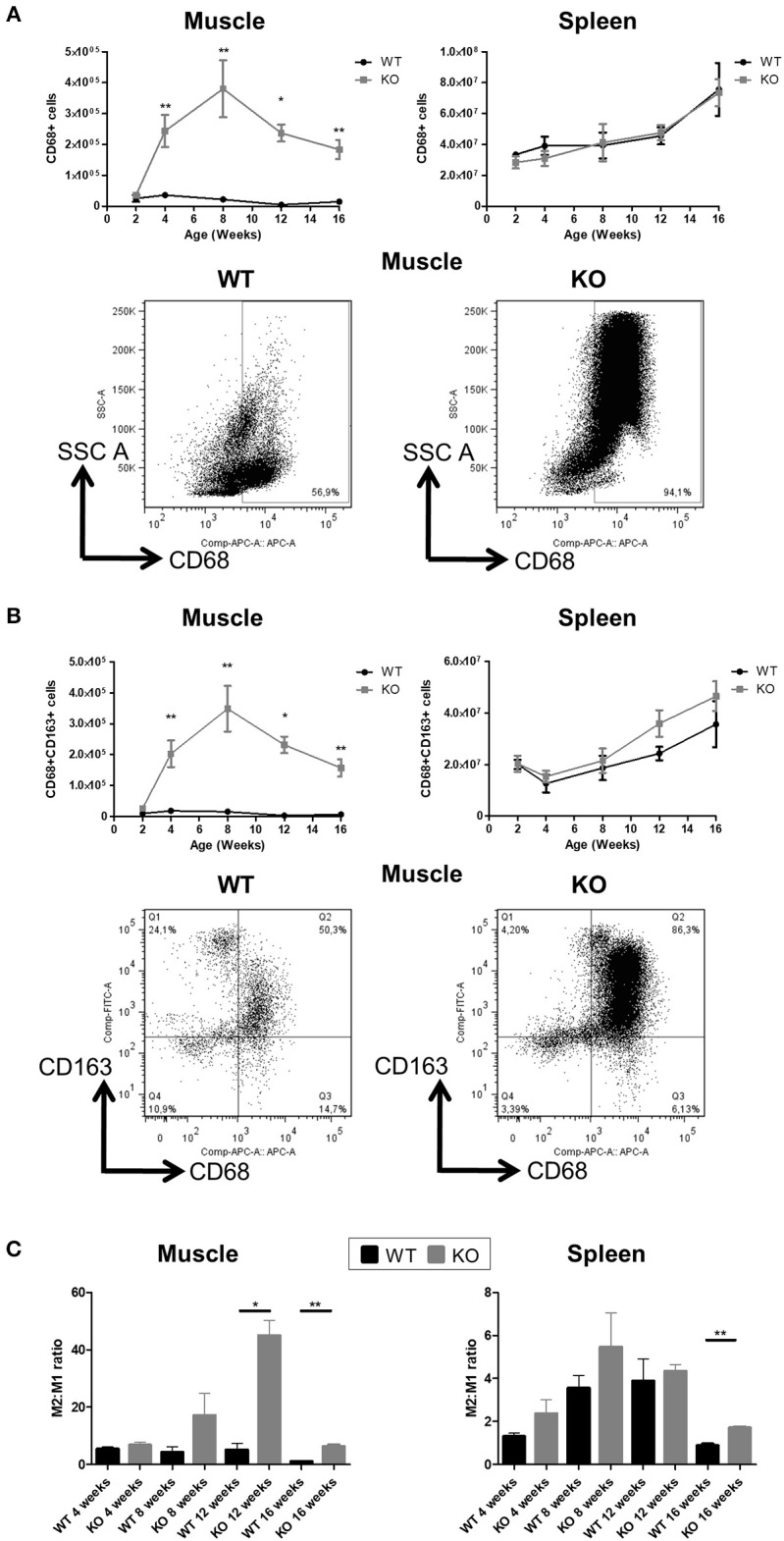
Number of macrophages in the skeletal muscle and spleen of *Dmd*^*mdx*^ rats. Cytofluorimetry of single-cell suspensions from hind limb muscles or spleen of WT or *Dmd*^*mdx*^ (KO) rats at the indicated time points. **(A)** Total number of macrophages (CD68^+^ cells) per gram of muscle (upper left panel) or whole spleen (upper right panel). Representative dot plots of high granularity macrophages using side scatter (SSC^high^) vs. CD68^+^ cells after gating on viable (negatively stained cells) CD45^+^ cells from muscle of WT or *Dmd*^*mdx*^ 12-week old rat**s** (lower panel). **(B)** Total number of viable CD68^+^CD163^+^ type 2 macrophages per gram of muscle (upper left panel) or whole spleen (upper right panel). Representative dot plots of viable CD68^+^CD163^+^ cells from muscle of WT or *Dmd*^*mdx*^ 12-week old rat**s** (lower panels). **(C)** Type 2 (CD68^+^CD163^+^) to type 1 macrophage (CD68^+^CD163^−^) ratios in muscle (left panel) or spleen (right panel) of WT (black) or *Dmd*^*mdx*^ (gray) rats. Data presented in **(A–C)** are given as mean ± SEM of 3, 6, 6, 7, and 8 (at 2, 4, 8, 12, and 16 weeks of age, respectively) *Dmd*^*mdx*^ rats and of 4, 6, 4, 3, and 4 (at 2, 4, 8, 12, and 16 weeks of age, respectively) WT rats. ^*^p < 0.05 and ^**^p < 0.01. Results were obtained from several experiments (*n* = 1, 2, 2, 3, and 3 at 2, 4, 8, 12, and 16 weeks of age, respectively) which were performed using all animal groups in each experiment.

About 90% of muscle CD45^+^ mononuclear leukocyte cells in *Dmd*^*mdx*^ rats were CD68^+^ (vs. ~60% in WT rats) (Figure 2A) and SIRPα (Supplementary Figure 1) at 2 weeks of age. CD68^+^ cells increased sharply at 4 weeks reaching their maximum at 8 weeks, and then decreased between 12 and 16 weeks of age. These cells showed significantly higher granularity as assessed by their SSC profile. Analysis of the M2 marker CD163 also showed a similar curve with an increase in CD68 expression (Figure 2B). In contrast, total CD68^+^ (Figure 2A), SIRPα (Supplementary Figure 1), or CD163^+^ macrophages (Figure 2B) in the spleen were not significantly different between *Dmd*^*mdx*^ and WT rats. The ratio of M2:M1 macrophages in the muscles of *Dmd*^*mdx*^ rats was similar at 4 weeks, increased at 8 weeks, and was significantly higher at 12 and 16 weeks of age. This ratio was low and remained constant in WT rat muscle (Figure 2C). The ratio of M2:M1 macrophages in the spleen increased over time but remained lower than that seen in muscles at all times. This ratio was similar for *Dmd*^*mdx*^ and WT rats, except at 16 weeks of age where *Dmd*^*mdx*^ rats showed a modest but significant increase compared to WT rats (Figure 2C).

**Figure 3:**
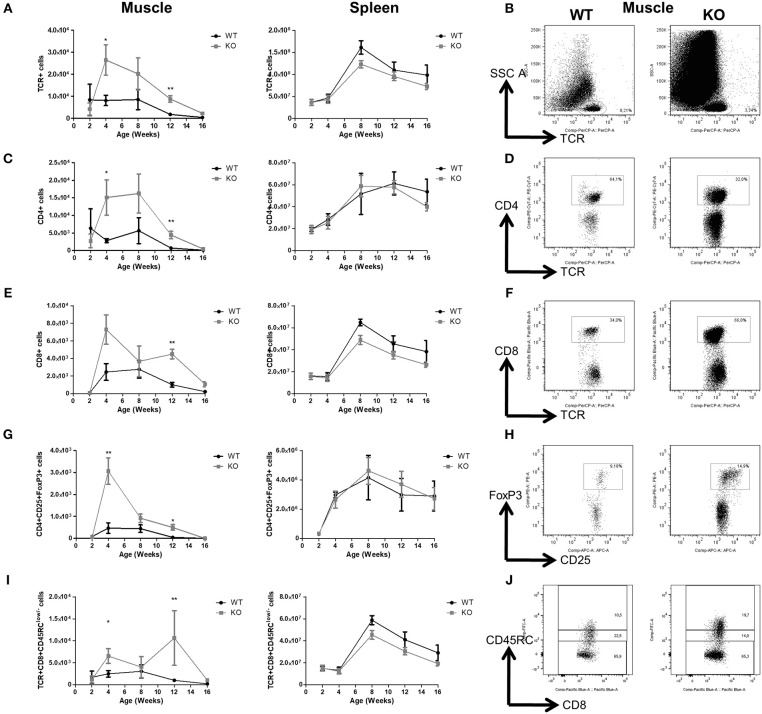
T cells in the skeletal muscle and spleen of *Dmd*^*mdx*^ rats. Hind limb muscles or spleen from WT or *Dmd*^*mdx*^ (KO) rats at the indicated time points were harvested, collagenase digested, and analyzed by cytofluorimetry. **(A)** Total numbers of viable CD45^+^TCR^+^ cells per gram of muscle (left panel) and whole spleen (right panel). **(B)** Representative dot plots of viable CD45^+^TCR^+^ cells from muscle of WT or *Dmd*^*mdx*^ 12-week old rats. **(C)** Total number of CD45^+^TCR^+^CD4^+^ cells per gram of muscle (left panel) and whole spleen (right panel). **(D)** Representative dot plots of WT and *Dmd*^*mdx*^ 12-week old rat muscle single-cell suspensions with gating on viable CD45^+^TCR^+^CD4^+^ cells. **(E)** Total number of TCR^+^CD8^+^ cells per gram of muscle (left panel) and whole spleen (right panel). **(F)** Representative dot plots of WT and *Dmd*^*mdx*^ 12-week old rat muscle single-cell suspensions with gating on viable CD45^+^TCR^+^CD8^+^ cells. **(G)** Total number of TCR^+^CD4^+^CD25^+^Foxp3^+^ cells per gram of muscle (left panel) and whole spleen (right panel). **(H)** Representative dot plots of WT and *Dmd*^*mdx*^ 12-week old rat muscle single-cell suspension with gating on viable CD45^+^TCR^+^CD4^+^CD25^+^Foxp3^+^ cells. **(I)** Total number of TCR^+^CD8^+^CD45RC^low/−^ cells per gram of muscle (left panel) and whole spleen (right panel). **(J)** Representative dot plots of WT or *Dmd*^*mdx*^ 12-week old rat muscle single-cell suspension with gating on viable CD45^+^TCR^+^CD8^+^CD45RC^low/−^ cells [corresponding to CD45^+^TCR^+^CD8^+^CD45RC^low^ cells (middle gate) + CD45^+^TCR^+^CD8^+^CD45RC^−^ cells (lower gate)]. Data presented in **(A,C,E,G,I)** are given as mean ± SEM of 3, 6, 10, 12, and 4 (at 2, 4, 8, 12, and 16 weeks of age, respectively) *Dmd*^*mdx*^ rats and 4, 5, 7, 7, and 4 (at 2, 4, 8, 12, and 16 weeks of age respectively) WT rats. Results were obtained from several experiments (*n* = 1, 2, 2, 3, and 3 at 2, 4, 8, 12, and 16 weeks of age, respectively) which were performed using all animal groups in each experiment. ^*^p < 0.05 and ^**^p < 0.01.

With regards to T cell subsets, total TCR^+^αβ (Figures 3A,B), CD4^+^ (Figures 3C,D), and CD8^+^ T cells (Figures 3E,F) in the muscles of *Dmd*^*mdx*^ rats increased sharply at 4 weeks of age, and then decreased at subsequent time points. The levels of these T cells were significantly higher at 4 and 12 weeks in *Dmd*^*mdx*^ compared to WT rats. Increased levels of Foxp3^+^CD4+ Tregs were also observed at 4 and 12 weeks (Figures 3G,H) in the muscles of *Dmd*^*mdx*^ vs. WT rats. CD8^+^ Tregs [defined as CD8^+^CD45RC^low/−^ T cells (22, 23)] were significantly increased at 4 and 12 weeks (Figures 3I,J).

In contrast, total TCR^+^CD4^+^ T cells and CD8^+^ T cells (Figures 3A–F) as well as total Foxp3^+^CD4^+^ Tregs and CD8^+^CD45RC^low/−^ Tregs in the spleen of *Dmd*^*mdx*^ and WT rats were comparable (Figures 3H,J).

B cells (CD45RA^+^ and CD45R^+^) and NK cells (CD161^high^) comprised between <2% and 3% of the total muscle leukocytes in both *Dmd*^*mdx*^ and WT rats, and levels in the spleen of *Dmd*^*mdx*^ and WT rats were similar (data not shown).

The majority of leukocytes in the muscles of *Dmd*^*mdx*^ rats were macrophages, and the M2:M1 ratio increased at 12 and 16 weeks of age. T cells, including CD8^+^ and CD4^+^ Tregs, showed a similar pattern to macrophages.

### Detection of Macrophages in Cardiac and Skeletal Muscle of *Dmd^*mdx*^* Rats by Immunohistology

**Figure 4:**
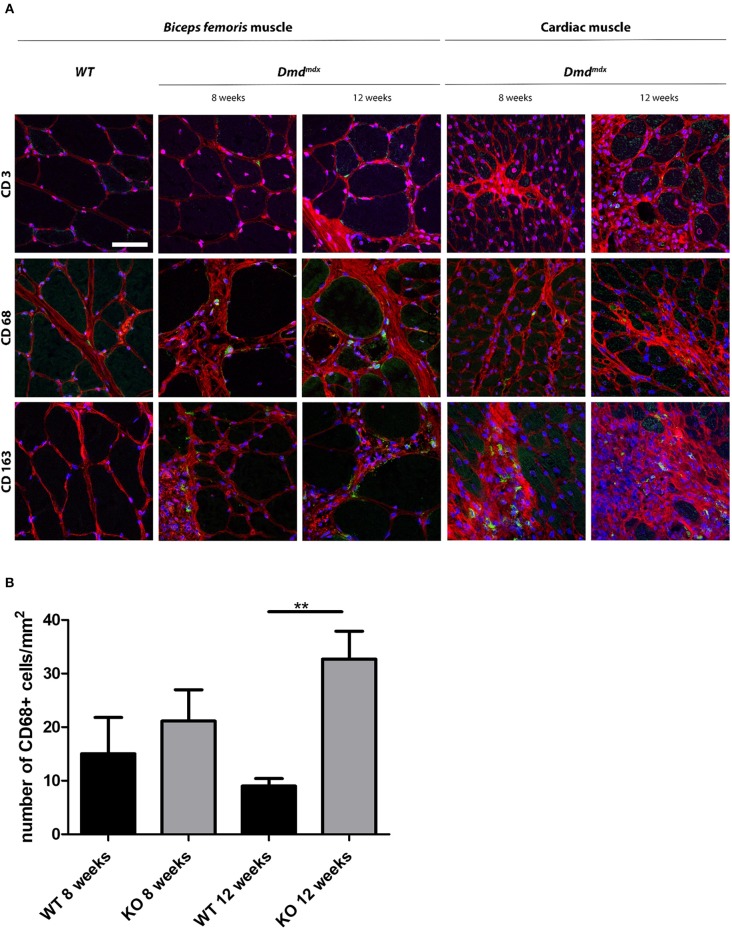
Immunohistochemical staining of leukocytes in skeletal and cardiac muscle of *Dmd*^*mdx*^ rats. **(A)** Skeletal (*Biceps femoris*) and cardiac muscle were harvested at 8 and 12 weeks of age from wild-type (WT) and *Dmd*^*mdx*^ (KO) rats. **(A)** Tissue sections were stained with Draq5 to label nuclei (blue), with wheat germ agglutinin for connective tissue (red), and with MAbs for detection of cells expressing CD3, CD68, or CD163 (green). Scale bar identical for all pictures: 100 μm. **(B)** Quantification of CD68^+^ macrophages. Ten fields were randomly chosen (WT 8 weeks *n* = 3; KO 8 weeks *n* = 4; WT 12 weeks *n* = 5; KO 12 weeks *n* = 12). ^**^p < 0.01.

Skeletal and cardiac muscle biopsies in 8- and 12-week old *Dmd*^*mdx*^ rats showed the presence of CD68^+^ and CD163^+^ macrophages and few CD3^+^ cells whereas only a few CD68^+^ macrophages were observed in the skeletal and cardiac muscles of WT rats (Figure 4A). At 12 weeks, the number of CD68^+^ cells in the cardiac muscle of *Dmd*^*mdx*^ rats was significantly increased (Figure 4B). CD163^+^ macrophages were notably numerous in the foci of mononuclear cell infiltrates in the cardiac muscle. As previously described (4), increased fibrosis (Figure 4A) occurs in the skeletal and cardiac muscle of *Dmd*^*mdx*^ rats from 4 weeks of age and is more severe at 8 weeks. In addition to these lesions, total creatinine kinase (CK) levels in serum were significantly increased in 4-, 8-, and 12-week *Dmd*^*mdx*^ rats, and returned to non-significantly different levels at week 16 (Supplementary Figure 3C, right panel).

These results indicate that infiltration of muscle by leukocytes was temporally associated with damaged muscle fibers and elevated CK serum levels.

### Inflammatory Mediators and Growth Factors in Leukocytes Infiltrating Muscle and Serum of *Dmd^*mdx*^* Rats

**Figure 5:**
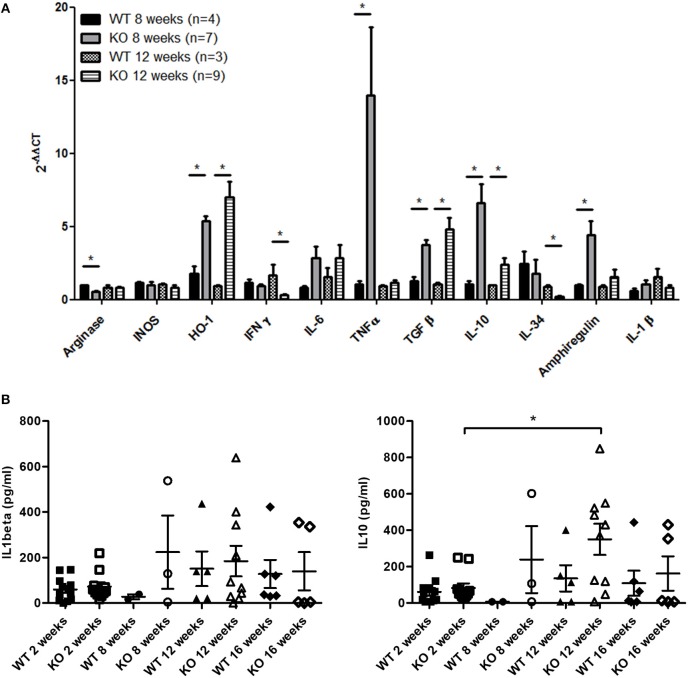
Inflammation markers and growth factors in skeletal muscle of *Dmd*^*mdx*^ rats. **(A)** Mononuclear cells from skeletal muscles were harvested at 8 and 12 weeks of age from wild-type (WT) and *Dmd*^*mdx*^ (KO) rats. Total RNA was extracted and mRNA levels for the indicated molecules were analyzed by quantitative RT-PCR. ^*^p < 0.05. **(B)** IL1β (left panel) and IL10 (right panel) levels in the sera of *Dmd*^*mdx*^ (*n* = 11, 3, 10, 5 at 2, 8, 12, and 16 weeks of age, respectively) or WT (*n* = 12, 2, 5, 6 at 2, 8, 12, and 16 weeks of age, respectively) rats.

Quantitative RT-PCR showed that the expression of TNFα in mononuclear cells from muscles of *Dmd*^*mdx*^ rats was particularly high compared to that in WT rats at 8 weeks. Heme oxygenase-1 (HO-1), TGFβ, IL-10, and the muscle trophic factor amphiregulin (12) were also significantly increased at 8 and/or 12 weeks (Figure 5A). IFNgamma expression decreased in 12-week old *Dmd*^*mdx*^ rats compared to WT rats of a similar age (Figure 5A). Levels of arginase and IL-34 were lower in *Dmd*^*mdx*^ rats compared to WT rats at weeks 8 and 12 (Figure 5A). IL-6, relaxin3, INOS, IL-1β, and indoleamine 2,3-dioxygenase (IDO) were detected at very low levels without any difference between groups (Figure 5A and data not shown).

Evaluation of cytokines in the sera of animals using a multiplex cytokine assay showed that IL-1beta and IL-10 were increased but were not significantly different between *Dmd*^*mdx*^ and WT rats (Figure 5B), and TNFα and IL-6 levels were undetectable (data not shown).

### Anti-CD45RC MAb Treatment Depletes CD45RC^high^ T Cells and Improves Skeletal Muscle Strength

Anti-CD45RC MAb treatment induces tolerance to organ transplantation and inhibits GVHD in rat models (15). Because CD45RC expression levels can differ between rat strains (24) and have not been reported for muscle, we first analyzed the distribution of CD45RC^high^ and CD45RC^low/−^ leukocytes within different leukocyte subsets in the muscle and spleen of *Dmd*^*mdx*^ and WT Sprague-Dawley rats.

The absolute number of TCR^+^CD8^+^CD45RC^low/−^ (Figures 3I,J) and CD45RC^high^ cells (Supplementary Figures 2A,B) within the CD8^+^ T cell population of *Dmd*^*mdx*^ rat muscle increased sharply and significantly at 4 weeks, remained elevated at 8 weeks, and decreased at 12 weeks to low levels compared to that seen in the muscles of WT rats. CD45RC^high^ and CD45RC^low/−^ cell numbers in the spleen were similar in *Dmd*^*mdx*^ and WT rats (Supplementary Figures 2A,B and Figure 3I).

Absolute numbers of CD45RC^low/−^ cells in the TCR^+^CD4^+^ cell population were significantly increased at 4 and 12 weeks in the muscle, but not in the spleen, of *Dmd*^*mdx*^ compared to WT rats (Supplementary Figures 2C,D). A similar pattern was observed for CD45RC^high^ cells although the higher cell numbers observed in the muscle of *Dmd*^*mdx*^ compared to WT rats were not significant (Supplementary Figures 2D,E).

Within the non-T cell compartment, which consisted mostly of macrophages, CD45RC^low/−^ sub-populations increased significantly at 4 weeks, remained elevated at 8 weeks, and decreased at 12 weeks (Supplementary Figures 2F,G) whereas there was a non-significant increase in TCR^−^CD45RC^high^ cells at 4 and 8 weeks in *Dmd*^*mdx*^ compared to WT rats (Supplementary Figures 2G,H). TCR^−^ cells in the spleen of *Dmd*^*mdx*^ and WT animals showed similar proportions of CD45RC^high^ and CD45RC^low/−^ cells (Supplementary Figures 2F–H).

**Figure 6:**
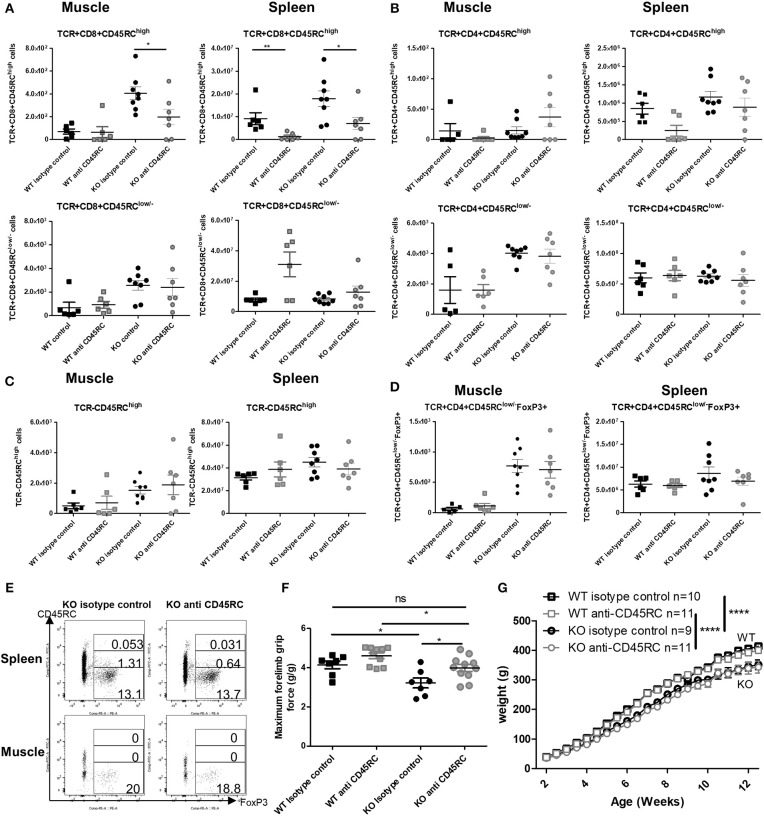
Effect of anti-CD45RC treatment on lymphoid cell populations, forelimb muscle strength, and animal growth. Hind limb muscles or spleen from WT or *Dmd*^*mdx*^ (KO) rats were harvested at 12 weeks of age, collagenase digested, and analyzed by cytofluorimetry. **(A)** Total number of viable CD45^+^TCR^+^CD8^+^CD45RC^high^ cells (upper panels) or viable CD45^+^TCR^+^CD8^+^CD45RC^low/−^ (lower panels) cells per gram of skeletal muscle (left panels) and for whole spleen (right panels). ^*^p < 0.05 and ^**^p < 0.01. **(B)** Total number of viable CD45^+^TCR^+^CD4^+^CD45RC^high^ cells (upper panels) or viable CD45^+^TCR^+^CD4^+^CD45RC^low/−^ (lower panels) cells per gram of skeletal muscle (left panels) and whole spleen (right panels). **(C)** Total number of viable CD45^+^TCR^−^ CD45RC^high^ cells per gram of skeletal muscle (left panels) and whole spleen (right panels). **(D)** Total number of viable CD45+TCR+CD45RC^low/−^FoxP3+ cells per gram of skeletal muscle (left panels) and whole spleen (right panels). **(E)** Representative dot plots of *Dmd*^*mdx*^ 12-week old rat spleen (upper panels) or muscle (lower panels) single-cell suspension, treated with anti CD45RC MAb or with a control MAb, with gating on viable CD45^+^TCR^+^CD4^+^CD45RChigh or low or -Foxp3^+^ cells. **(F)** Muscle strength in *Dmd*^*mdx*^ rats after treatment with an anti-CD45RC MAb. Wild-type (WT) or *Dmd*^*mdx*^ rats received intraperitoneal injections of the anti-rat CD45RC MAb (clone OX22, 1.5 mg/kg, every 3.5 days) or isotype control MAb (clone 3G8, 1.5 mg/kg, every 3.5 days) from week 2 to 12 when muscle strength was analyzed using a grip test. Each point represents a single animal analyzed in two different experiments. ^*^p < 0.05; ns, not statistically significant. Results were obtained from several experiments which were performed using all animal groups in each experiment and analyzed by a blinded operator. **(G)** Weight curves for animal growth were determined serially. ^****^p < 0.0001 for the whole data in the curve between *Dmd*^*mdx*^ and WT rats for both treatments but no difference between *Dmd*^*mdx*^ rats treated with anti-CD45RC vs. isotype control.

By 12 weeks of age, anti-CD45RC MAb treatment had significantly depleted CD8^+^CD45RC^high^ T cells in both the muscle and spleen of *Dmd*^*mdx*^ rats and in the spleen of WT rats and resulted in a predominance of CD8^+^CD45RC^low/−^ cells (Figure 6A). Numbers of CD4^+^CD45RC^high^ T cells in the spleen were decreased but did not reach statistical significance (Figure 6B). CD4^+^CD45RC^low/−^ (Figure 6B) and FoxP3^+^ CD4+CD45RC^low/−^ T cells (Figures 6D,E) remained unchanged in both the muscle and spleen. As observed in the transplantation models outlined above, other TCR^−^CD45RC^high^ leukocytes such as B cells were not depleted by anti-CD45RC treatment (Figure 6C). There was no difference between *Dmd*^*mdx*^ rats treated or not treated with anti-CD45RC in terms of inflammatory markers analyzed by quantitative RT-PCR in the muscle (data not shown).

As previously reported, muscle strength at 12 weeks of age using the grip test (4), *Dmd*^*mdx*^ rats showed a significant 30% reduction in forelimb peak strength compared to WT littermates (Figure 6F). Treatment with anti-CD45RC MAb significantly improved peak muscle strength in *Dmd*^*mdx*^ rats compared to control *Dmd*^*mdx*^ animals (Figure 6F). Furthermore, values for *Dmd*^*mdx*^ rats treated with anti-CD45RC MAb were indistinguishable from those for littermate WT controls (Figure 6F). This was despite *Dmd*^*mdx*^ rats showing significantly lower strength compared to WT animals treated with anti-CD45RC. The latter was however due to a slight, non-significant increase in the muscle strength of WT animals treated with anti-CD45RC vs. WT isotype control-treated animals (Figure 6F). Fatigue after repeated muscle effort, was not ameliorated by anti-CD45RC treatment (data not shown). The weight gain was significantly lower in *Dmd*^*mdx*^ compared to WT animals; this was not modified by anti-CD45RC treatment (Figure 6G). Skeletal muscle fibrosis (Supplementary Figures 3A,B), or serum CK levels (Supplementary Figure 3C) were not modified by anti-CD45RC treatment.

Thus, anti-CD45RC treatment resulted in increased peak muscle strength in *Dmd*^*mdx*^ rats that was associated with the depletion of T CD8^+^CD45RC^high^ cells.

### Prednisolone Improves Skeletal Muscle Strength but Has Secondary Effects

**Figure 7:**
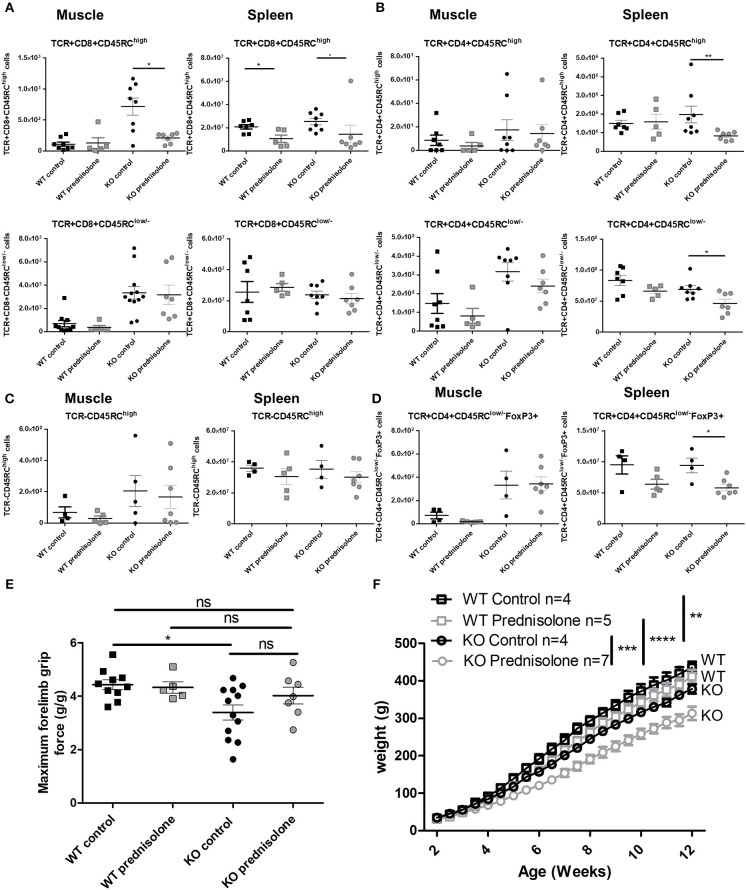
Effect of prednisolone treatment on lymphoid cell populations and forelimb muscle strength. Wild-type (WT) or *Dmd*^*mdx*^ (KO) rats received intraperitoneal injections of prednisolone (0.5 mg/kg, 5 days per week) or NaCl from week 2 up to 12. **(A)** Hind limb muscles or spleen from WT or *Dmd*^*mdx*^ were harvested, collagenase digested, and analyzed by cytofluorimetry. Total number of viable CD45^+^TCR^+^CD8^+^CD45RC^high^ cells (upper panels) or viable CD45^+^TCR^+^CD8^+^CD45RC^low/−^ (lower panels) cells per gram of muscle (left panels) and whole spleen (right panels). ^*^p < 0.05. **(B)** Total number of viable CD45^+^TCR^+^CD4^+^CD45RC^high^ cells (upper panels) or viable CD45^+^TCR^+^CD4^+^CD45RC^low/−^ (lower panels) cells per gram of muscle (left panels) and whole spleen (right panels). ^**^p < 0.01. **(C)** Total number of viable CD45^+^TCR^−^ CD45RC^high^ cells per gram of skeletal muscle (left panels) and for whole spleen (right panels). **(D)** Total number of viable CD45+TCR+CD45RC^low/−^FoxP3+ cells per gram of skeletal muscle (left panels) and whole spleen (right panels). ^*^p < 0.05. **(E)** Muscle strength was analyzed using a grip test. Each point represents a single animal analyzed in two different experiments. ^*^p < 0.05. ns, not statistically significant. Results were obtained from several experiments which were performed using all animal groups in each experiment. **(F)** Weight curves for animal growth were determined serially. ^**^p < 0.01 and ^****^p < 0.0001 for *Dmd*^*mdx*^ and WT with NaCl and prednisolone but importantly ^***^p < 0.001 between *Dmd*^*mdx*^ rats NaCl vs. prednisolone.

Because CS are standard treatment for DMD patients (7), we analyzed the effect of prednisolone in *Dmd*^*mdx*^ rats. A significant decrease in CD8^+^CD45RC^high^ T cells in the muscle and spleen of *Dmd*^*mdx*^ rats treated with prednisolone and in the spleen of similarly treated WT rats was observed at 12 weeks of age (Figure 7A), whilst the number of CD8^+^CD45RC^low/−^ T cells remained unchanged (Figure 7A). CD4^+^CD45RC^high^ T cells were significantly decreased in the spleen but not in the muscle (Figure 7B). CD4^+^CD45RC^low/−^ T cells were also decreased in the spleen but not in the muscle (Figure 7B). TCR^−^CD45RC^high^ leukocytes such as macrophages and B cells were not depleted by prednisolone treatment (Figure 7C). TCR^+^CD4^+^CD45RC^low/−^FoxP3^+^ were significantly decreased in the spleen but not in the muscle of prednisolone-treated *Dmd*^*mdx*^ rats (Figure 7D).

Treatment with prednisolone significantly recovered muscle strength in *Dmd*^*mdx*^ rats to a level similar to that seen in WT animals and with a tendency compared to control untreated *Dmd*^*mdx*^ rats (Figure 7E). Prednisolone-treated *Dmd*^*mdx*^ rats showed a severe (25%) growth reduction compared to WT and NaCl-treated *Dmd*^*mdx*^ rats (Figure 7F). Prednisolone had no effect on the growth of WT animals (Figure 7F). Muscle tissue fibrosis (Supplementary Figures 3A,B) and serum CK levels (Supplementary Figure 3C) were not modified by prednisolone treatment.

When comparing the group of *Dmd*^*mdx*^ rats treated with anti-CD45RC to those treated with prednisolone, the peak force and CK were comparable whereas growth was significantly higher in the anti-CD45RC group (Supplementary Figure 4).

In summary, compared to anti-CD45RC treatment, prednisolone increased muscle strength but decreased immune cell populations and had a strong negative effect on the growth of *Dmd*^*mdx*^ animals.

### Presence of T CD45RC^high^ Cells in Skeletal Muscles and Blood of DMD Patients

**Figure 8:**
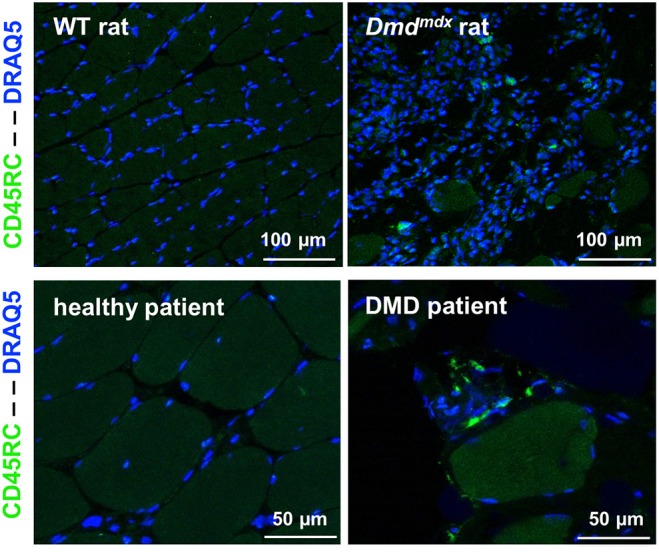
CD45RC^+^ cells in rat and human dystrophin-deficient skeletal muscles. Skeletal muscle samples from rats (*Biceps femoris*) and humans (*Paravertebralis*), either from dystrophin-deficient individuals (*n* = 2) or those without muscle pathology (*n* = 2). Pictures are representative images of frozen tissue sections stained with Draq5 to label nuclei and with anti-rat or human anti-CD45RC MAbs (green).

CD45RC^high^ and CD45RC^low/−^ cells in the CD4^+^ or CD8^+^ T cell compartments in the blood of DMD patients were present in comparable proportions to that seen in age-matched individuals hospitalized for pathologies not involving the immune system or neuromuscular diseases (Supplementary Figure 5). The presence of CD45RC positive cells was confirmed through muscle biopsy in DMD patients. As most tissue macrophages are CD45RC^−^, these CD45RC^+^ cells are likely T cells. These were not observed in muscle samples from normal individuals (Figure 8).

## Discussion

DMD patients and *mdx* mouse skeletal muscles are infiltrated by different leukocyte types that produce mediators to either promote or protect against disease evolution (6). Apart from inflammation and innate immune responses, adaptive immune responses including anti-dystrophin T cells and Treg cells are also present in DMD patients (9, 11) and *mdx* mice (6, 12, 13). CS, one of the only standard treatments received by DMD patients, may prolong ambulation by about 2 years. Nevertheless, increases in muscular strength responses are variable, incomplete, and always associated with serious side effects (7, 8). The precise mechanism of CS action in DMD patients is poorly defined; however, anti-inflammatory effects are likely very important (7, 8). Thus, unmet clinical needs exist in the treatment of the inflammatory and immune effects caused by dystrophin deficiency. It is also very likely that in the future these immunotherapies will be coupled to gene and cell therapies in order to inhibit immune responses against the vectors, transgene products, or antigenic cellular products.

Whilst the *mdx* mouse is a very useful model, it fails to reproduce key DMD patient symptoms such as muscle weakness (3). Several immunotherapies, including intravenous immunoglobulin (22), anti-TNFα antibodies (23), IL-6 blocking Abs (24), tranilast (25), heme oxygenase-1 (HO-1) inducers (26), IL-1 receptor antagonists (27), and IL-2 complexes to amplify CD4^+^ Tregs (13), have been successful in treating *mdx* mice; however, their potential effect on DMD patients is unclear.

Skeletal and cardiac muscle weakness occurs at early time points in *Dmd*^*mdx*^ rats, and skeletal and cardiac muscle lesions resemble those observed in DMD patients (4, 5). In this study, we showed that mononuclear cells infiltrate *Dmd*^*mdx*^ rat skeletal and cardiac muscles between 2 and 4 weeks of age, reach a maximum between 8 and 12 weeks, and decrease by 16 weeks of age. Most of these mononuclear cells were CD68^+^ and SIRPα^+^ macrophages, and the proportion of M2 CD163^+^ cells increased over time. Macrophages appear early in both *mdx* mice (2 weeks) and DMD patients (2 years of age) (28). M2 macrophages have been shown to play protective and regenerative roles in the early stages of disease in *mdx* mice (6). CD4^+^ and CD8^+^ T cells, including Tregs, were also increased in the muscles of *Dmd*^*mdx*^ rats compared to controls. Lesions in the muscular fibers were assessed by serum CK levels and the results paralleled the leukocyte infiltration kinetics. Normal levels of serum CK were seen at 2 weeks of age, with a peak between 4 and 8 weeks and a subsequent decrease. This may reflect the occurrence of a more pronounced immune response at early rather than later time points.

Mononuclear cells from *Dmd*^*mdx*^ rats expressed increased levels of cytokine transcripts including TNFα compared to controls at 8 and/or 12 weeks of age. These cytokines, which are increased in DMD patients and *mdx* mice, are potential immunotherapy targets (29). Anti-TNFα treatment reduces early muscle damage in *mdx* mice (23) and could be targeted in *Dmd*^*mdx*^ rats in the future. Several anti-inflammatory molecules, such as HO-1, IL-10, and TGFβ as well as the muscle trophic factor amphiregulin (12), were also expressed, most likely as a response to inflammation and ongoing immune responses, as has been previously described in *mdx* mice and DMD patients (6). TGFβ plays a dual role in *mdx* mice, as early neutralization of this cytokine has been shown to decrease fibrosis but to increase T cell infiltration and inflammation (30).

We have recently shown that treatment with an anti-CD45RC MAb in a rat model of heart allograft rejection could induce permanent allograft acceptance (15). Furthermore, anti-CD45RC MAb treatment prevented GVHD in immune-humanized immunodeficient NSG mice (15). Anti-CD45RC depleted T cells that were CD45RC^high^; these included naïve T cells, precursors of Th1 cells, and T effector memory cells including TEMRA cells. CD8^+^ and CD4^+^ Tregs in both rats and humans are CD45RC^low/−^ (14, 31) and were thus spared. The latter were specific for donor alloantigens and could induce allograft tolerance in newly grafted irradiated recipients following adoptive cell transfer.

Treatment of *Dmd*^*mdx*^ rats with anti-CD45RC MAbs could eliminate CD45RC^high^ effector T cells and their precursors and enrich CD45RC^low/−^ Tregs. These could then inhibit immune responses by CD45RC^high^ and promote tissue repair and homeostasis by CD45RC^low/−^, as has been described for CD4^+^ Tregs in both muscle (12) and adipose tissue (32). We show that treatment of *Dmd*^*mdx*^ rats with anti-CD45RC improved muscle strength to levels similar to that seen in WT littermates, and that this was associated with depletion of CD8^+^ CD45RC^high^ T cells at 12 weeks of age. CD4^+^ CD45RC^high^ T effector cells decreased at this time point; however, this was not statistically significant. CD45RC^low/−^ CD8^+^ or CD4^+^ Tregs did not increase in *Dmd*^*mdx*^ rats; this was similar to rats tolerant to transplanted organs following anti-CD45RC MAb treatment but their alloantigen suppressive activity was increased (15). Whether CD8^+^ or CD4^+^ Tregs plays a role in the amelioration of muscle strength observed in these animals remains to be explored.

CS treatment of *Dmd*^*mdx*^ rats increased muscular strength associated with a decrease in CD8^+^CD45RC^high^ T cells in muscle and a more widespread decrease of CD4^+^CD45RC^high^ and CD4^+^CD45RC^low/−^ cells in the spleen. DMD patients show CD45RC^high^ cells in muscle and CD4^+^ or CD8^+^CD45RC^high^ T cells in blood despite treatment with CS; these cells decreased in the muscle and spleen of CS-treated rats. DMD patients treated with CS also show a variable and time-limited increase in muscle strength (6) and decreased dystrophin-specific T cells (11).

Secondary effects of steroids were observed in *Dmd*^*mdx*^ rats; animals treated with anti-CD45RC did not show obvious clinical abnormalities or weight loss. Anti-CD45RC treatment could result in similar muscle improvement as that seen with corticosteroids but without the side effects. Nevertheless, Vamorolone, a new anti-inflammatory drug which acts through the glucocorticoid receptor, has been shown to normalize maximal forelimb strength in the *mdx* model without the side effects seen with prednisolone (33). It also had a favorable profile in a recent Phase II clinical trial (34). Generalized immunosuppression could be a potential side effect of anti-CD45RC treatment; however, we showed that rats treated with anti-CD45RC could mount normal primary immune responses to new antigens as well as memory immune responses after secondary immunization (15).

Anti CD45RA (29), CD45RO/B (35), and CD45RB (36) MAbs have been used to treat organ rejection and/or GVHD. None of these however, have been used as an isolated treatment in animal models of DMD or muscle lesions. Up to 50–90% of CD8^+^ and CD4^+^ Tregs are CD45RA^high^ and CD45RB^high^ (15). Thus, treatment with anti-CD45RC clearly targets different cell populations and is likely more favorable as it preserves Tregs. Although, depletion of total CD4^+^ or CD8^+^ cells in *mdx* mice ameliorates the histopathology (37) this would not be a potential treatment for DMD patients and no other MAb-based tolerizing treatment used in organ transplantation, GVHD or autoimmunity, such as anti-CD3, anti-CD127, anti-CD28, has been previously used in DMD models. Thus, treatment with anti-CD45RC is novel and could stimulate further studies using tolerizing strategies in DMD patients.

## Data Availability

The datasets generated for this study are available on request to the corresponding author.

## Ethics Statement

The studies involving human participants were reviewed and approved by MESR/DC-2010-1199, CPP/29/10, and MESR DC-2011-1399. Written informed consent to participate in this study was provided by the participants' legal guardian/next of kin. The animal study was reviewed and approved by CEEA-PdL-10792 and CEEA-PdL-8986.

## Author Contributions

IA designed the research, analyzed data, obtained funding, and wrote the article. L-HO and SR wrote the article, designed the research, performed research, and analyzed data. AL, TL, LT, VC, LB, and CB performed research and analyzed data. RJ, KR, YP, AC, CG-LG, and NV contributed vital reagents. TL, RJ, CB, KR, CH, and CG critically reviewed the manuscript.

### Conflict of Interest Statement

IA and CG have registered a patent on the use of anti-CD45RC for treatment of Duchenne's disease. The remaining authors declare that the research was conducted in the absence of any commercial or financial relationships that could be construed as a potential conflict of interest.

## Abbreviations

DMD: Duchenne muscular dystrophy
CS: corticosteroids
MAb: monoclonal antibody
GVHD: graft vs. host disease
CK: creatinine phosphokinase
WT: wild type
KO: knockout
IDO: indoleamine 2, 3-dioxygenase
HO-1: heme oxygenase-1
NSG: NOD *Scid* Gamma.
