# Supplementary material for: Immunophenotype of a Rat Model of Duchenne's Disease and Demonstration of Improved Muscle Strength After Anti-CD45RC Antibody Treatment
Source: Front Immunol. 2019 Sep 9;10:2131. doi: 10.3389/fimmu.2019.02131 (PMC6746111; doi:10.3389/fimmu.2019.02131)
Supplement: Supplementary file 1 [file Presentation_1.ppt]

## Slide 1
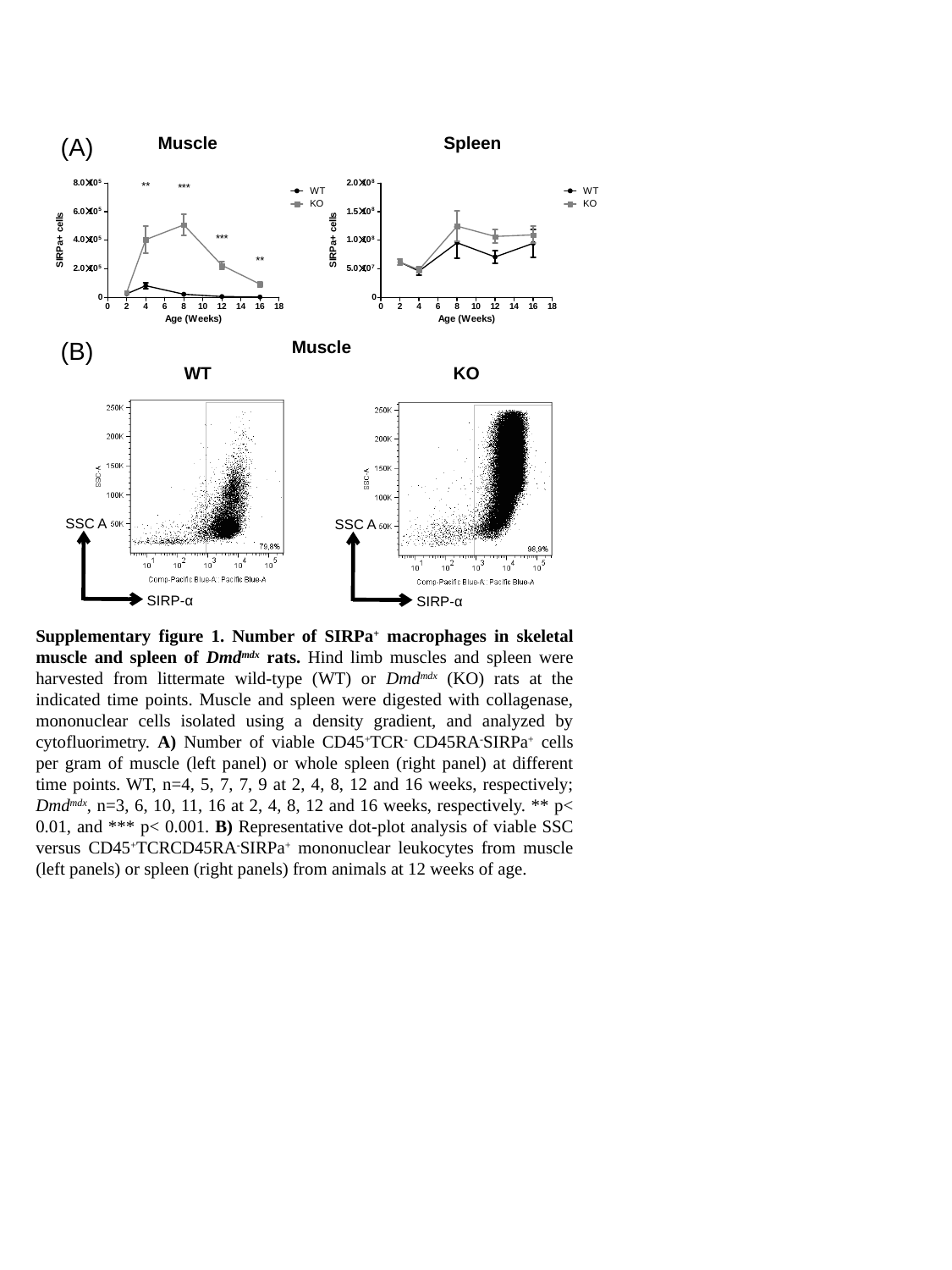

(A)
Muscle
Spleen
(B)
Muscle
WT
KO
SSC A
SSC A
SIRP-α
SIRP-α
Supplementary figure 1. Number of SIRPa+ macrophages in skeletal muscle and spleen of Dmdmdx rats. Hind limb muscles and spleen were harvested from littermate wild-type (WT) or Dmdmdx (KO) rats at the indicated time points. Muscle and spleen were digested with collagenase, mononuclear cells isolated using a density gradient, and analyzed by cytofluorimetry. A) Number of viable CD45+TCR- CD45RA-SIRPa+ cells per gram of muscle (left panel) or whole spleen (right panel) at different time points. WT, n=4, 5, 7, 7, 9 at 2, 4, 8, 12 and 16 weeks, respectively; Dmdmdx, n=3, 6, 10, 11, 16 at 2, 4, 8, 12 and 16 weeks, respectively. ** p< 0.01, and *** p< 0.001. B) Representative dot-plot analysis of viable SSC versus CD45+TCRCD45RA-SIRPa+ mononuclear leukocytes from muscle (left panels) or spleen (right panels) from animals at 12 weeks of age.

## Slide 2
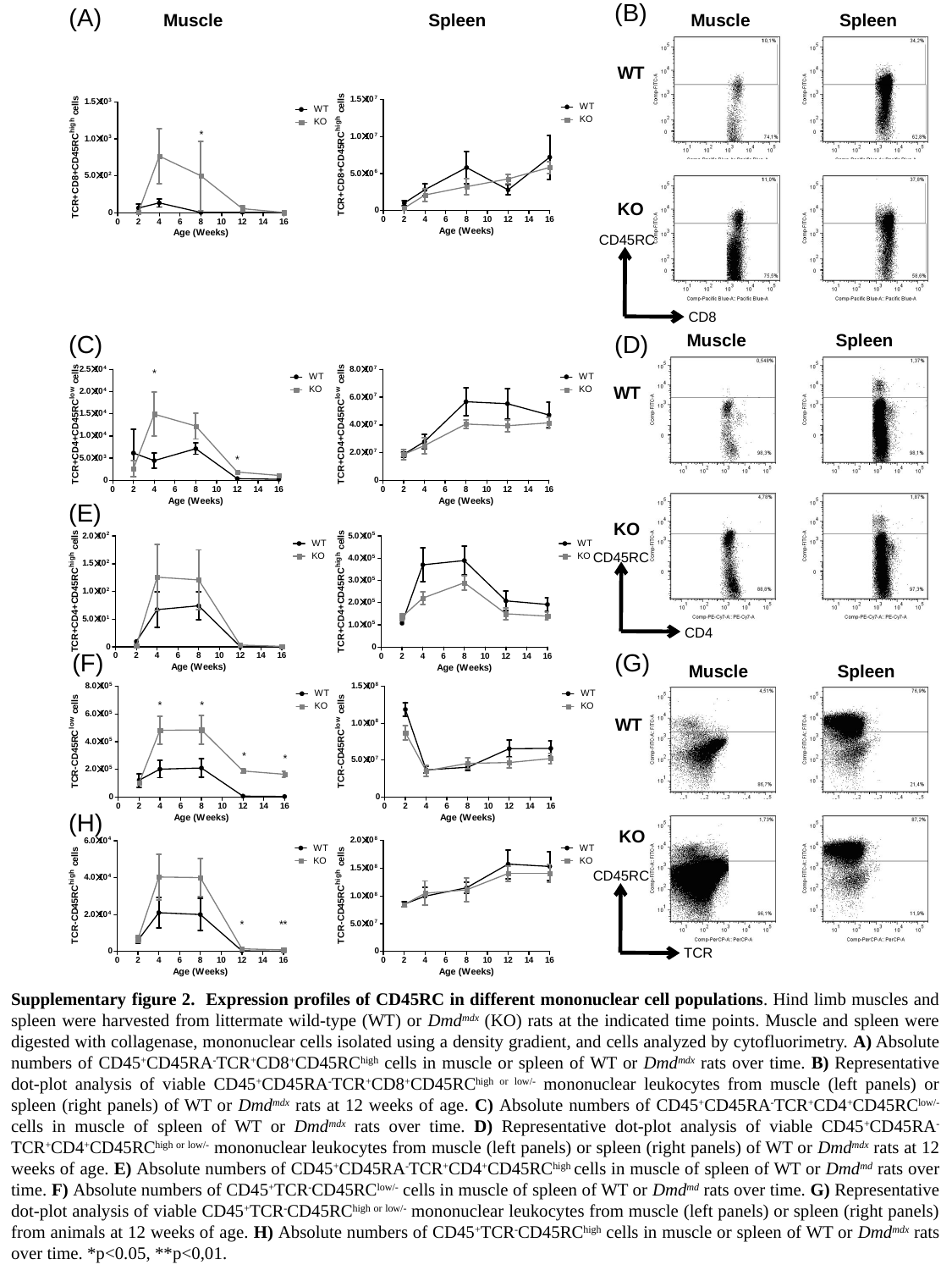

(B)
(A)
Muscle
Spleen
Muscle
Spleen
WT
KO
CD45RC
CD8
(C)
(D)
Muscle
Spleen
WT
(E)
KO
CD45RC
CD4
(F)
(G)
Muscle
Spleen
WT
(H)
KO
CD45RC
TCR
Supplementary figure 2. Expression profiles of CD45RC in different mononuclear cell populations. Hind limb muscles and spleen were harvested from littermate wild-type (WT) or Dmdmdx (KO) rats at the indicated time points. Muscle and spleen were digested with collagenase, mononuclear cells isolated using a density gradient, and cells analyzed by cytofluorimetry. A) Absolute numbers of CD45+CD45RA-TCR+CD8+CD45RChigh cells in muscle or spleen of WT or Dmdmdx rats over time. B) Representative dot-plot analysis of viable CD45+CD45RA-TCR+CD8+CD45RChigh or low/- mononuclear leukocytes from muscle (left panels) or spleen (right panels) of WT or Dmdmdx rats at 12 weeks of age. C) Absolute numbers of CD45+CD45RA-TCR+CD4+CD45RClow/- cells in muscle of spleen of WT or Dmdmdx rats over time. D) Representative dot-plot analysis of viable CD45+CD45RA-TCR+CD4+CD45RChigh or low/- mononuclear leukocytes from muscle (left panels) or spleen (right panels) of WT or Dmdmdx rats at 12 weeks of age. E) Absolute numbers of CD45+CD45RA-TCR+CD4+CD45RChigh cells in muscle of spleen of WT or Dmdmd rats over time. F) Absolute numbers of CD45+TCR-CD45RClow/- cells in muscle of spleen of WT or Dmdmd rats over time. G) Representative dot-plot analysis of viable CD45+TCR-CD45RChigh or low/- mononuclear leukocytes from muscle (left panels) or spleen (right panels) from animals at 12 weeks of age. H) Absolute numbers of CD45+TCR-CD45RChigh cells in muscle or spleen of WT or Dmdmdx rats over time. *p<0.05, **p<0,01.

## Slide 3
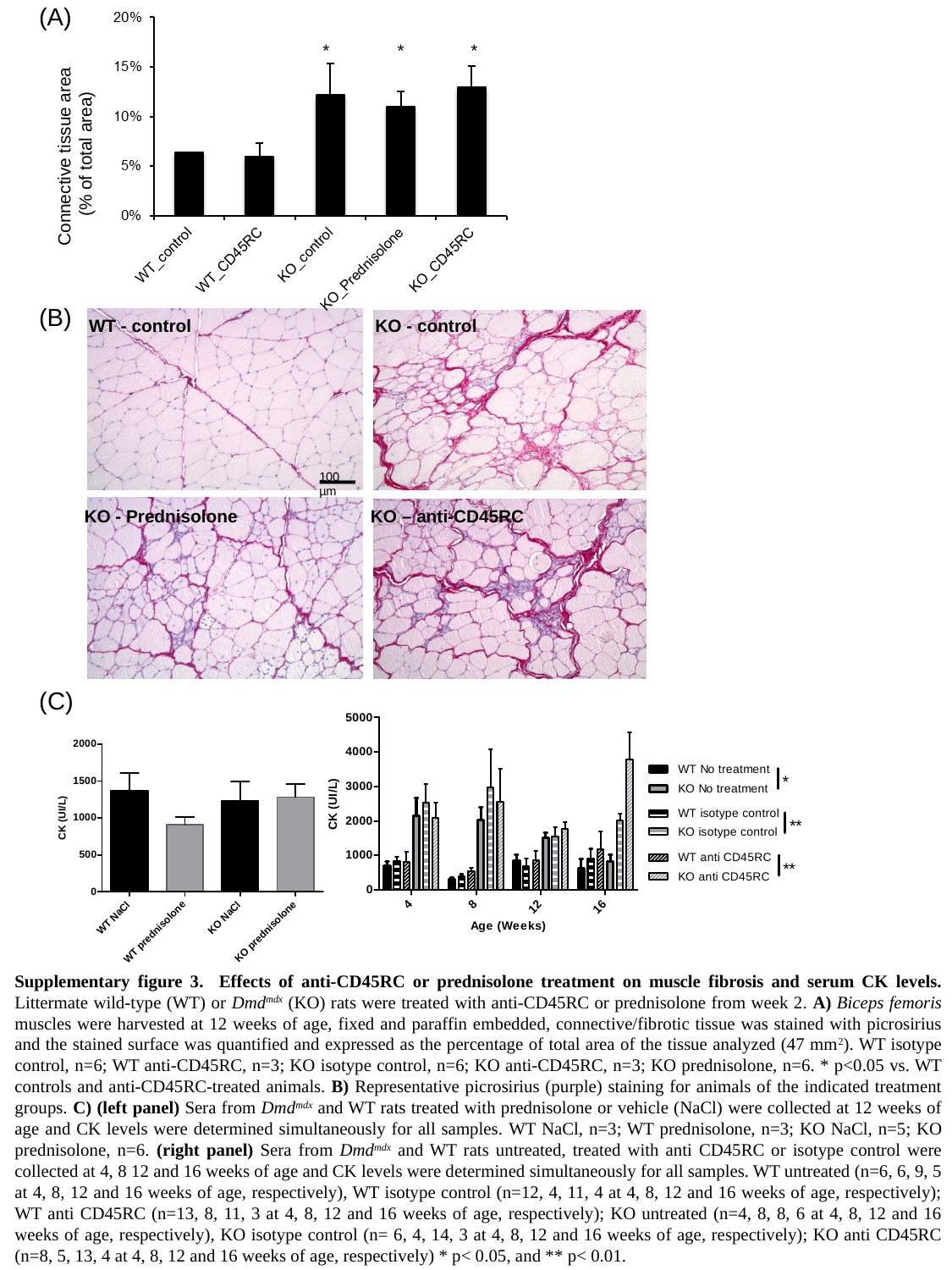

(A)
*
*
*
Connective tissue area
(% of total area)
(B)
WT - control
KO - control
KO - Prednisolone
KO – anti-CD45RC
100 µm
(C)
Supplementary figure 3. Effects of anti-CD45RC or prednisolone treatment on muscle fibrosis and serum CK levels. Littermate wild-type (WT) or Dmdmdx (KO) rats were treated with anti-CD45RC or prednisolone from week 2. A) Biceps femoris muscles were harvested at 12 weeks of age, fixed and paraffin embedded, connective/fibrotic tissue was stained with picrosirius and the stained surface was quantified and expressed as the percentage of total area of the tissue analyzed (47 mm2). WT isotype control, n=6; WT anti-CD45RC, n=3; KO isotype control, n=6; KO anti-CD45RC, n=3; KO prednisolone, n=6. * p<0.05 vs. WT controls and anti-CD45RC-treated animals. B) Representative picrosirius (purple) staining for animals of the indicated treatment groups. C) (left panel) Sera from Dmdmdx and WT rats treated with prednisolone or vehicle (NaCl) were collected at 12 weeks of age and CK levels were determined simultaneously for all samples. WT NaCl, n=3; WT prednisolone, n=3; KO NaCl, n=5; KO prednisolone, n=6. (right panel) Sera from Dmdmdx and WT rats untreated, treated with anti CD45RC or isotype control were collected at 4, 8 12 and 16 weeks of age and CK levels were determined simultaneously for all samples. WT untreated (n=6, 6, 9, 5 at 4, 8, 12 and 16 weeks of age, respectively), WT isotype control (n=12, 4, 11, 4 at 4, 8, 12 and 16 weeks of age, respectively); WT anti CD45RC (n=13, 8, 11, 3 at 4, 8, 12 and 16 weeks of age, respectively); KO untreated (n=4, 8, 8, 6 at 4, 8, 12 and 16 weeks of age, respectively), KO isotype control (n= 6, 4, 14, 3 at 4, 8, 12 and 16 weeks of age, respectively); KO anti CD45RC (n=8, 5, 13, 4 at 4, 8, 12 and 16 weeks of age, respectively) * p< 0.05, and ** p< 0.01.

## Slide 4
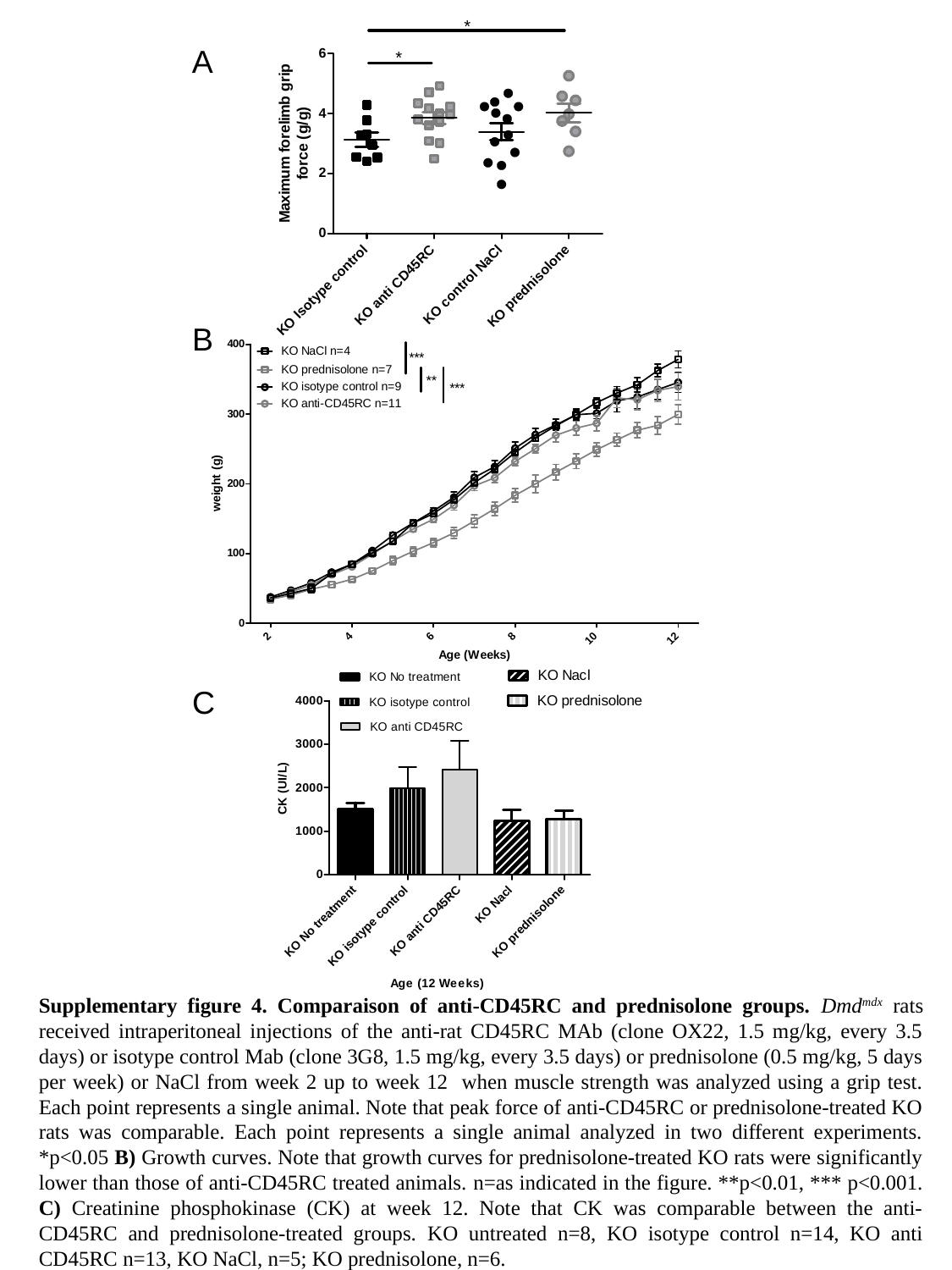

A
B
C
Supplementary figure 4. Comparaison of anti-CD45RC and prednisolone groups. Dmdmdx rats received intraperitoneal injections of the anti-rat CD45RC MAb (clone OX22, 1.5 mg/kg, every 3.5 days) or isotype control Mab (clone 3G8, 1.5 mg/kg, every 3.5 days) or prednisolone (0.5 mg/kg, 5 days per week) or NaCl from week 2 up to week 12 when muscle strength was analyzed using a grip test. Each point represents a single animal. Note that peak force of anti-CD45RC or prednisolone-treated KO rats was comparable. Each point represents a single animal analyzed in two different experiments. *p<0.05 B) Growth curves. Note that growth curves for prednisolone-treated KO rats were significantly lower than those of anti-CD45RC treated animals. n=as indicated in the figure. **p<0.01, *** p<0.001. C) Creatinine phosphokinase (CK) at week 12. Note that CK was comparable between the anti-CD45RC and prednisolone-treated groups. KO untreated n=8, KO isotype control n=14, KO anti CD45RC n=13, KO NaCl, n=5; KO prednisolone, n=6.

## Slide 5
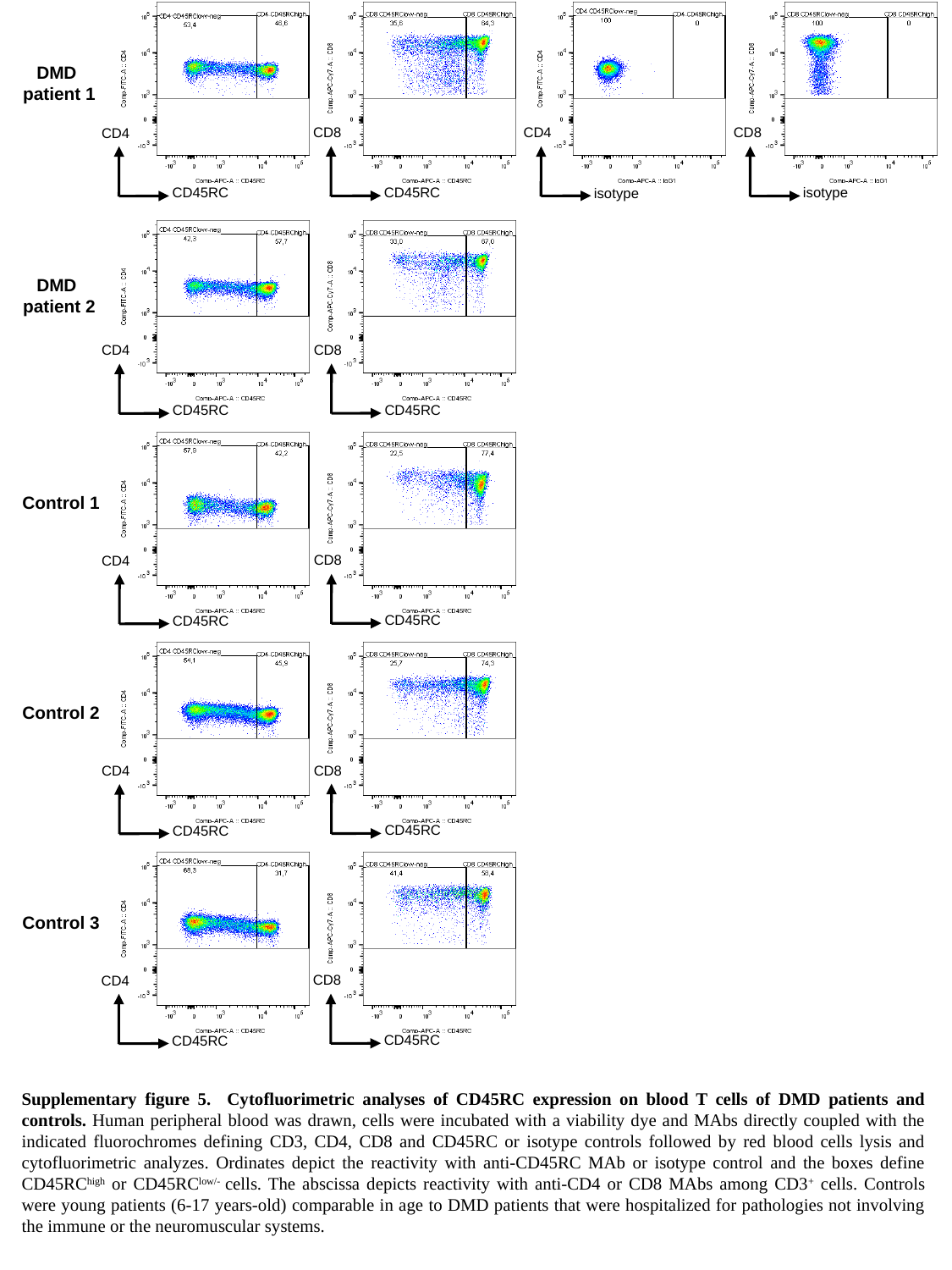

DMD
patient 1
CD8
CD4
CD8
CD4
CD45RC
CD45RC
isotype
isotype
DMD
patient 2
CD8
CD4
CD45RC
CD45RC
Control 1
CD8
CD4
CD45RC
CD45RC
Control 2
CD8
CD4
CD45RC
CD45RC
Control 3
CD8
CD4
CD45RC
CD45RC
Supplementary figure 5. Cytofluorimetric analyses of CD45RC expression on blood T cells of DMD patients and controls. Human peripheral blood was drawn, cells were incubated with a viability dye and MAbs directly coupled with the indicated fluorochromes defining CD3, CD4, CD8 and CD45RC or isotype controls followed by red blood cells lysis and cytofluorimetric analyzes. Ordinates depict the reactivity with anti-CD45RC MAb or isotype control and the boxes define CD45RChigh or CD45RClow/- cells. The abscissa depicts reactivity with anti-CD4 or CD8 MAbs among CD3+ cells. Controls were young patients (6-17 years-old) comparable in age to DMD patients that were hospitalized for pathologies not involving the immune or the neuromuscular systems.

## Slide 6
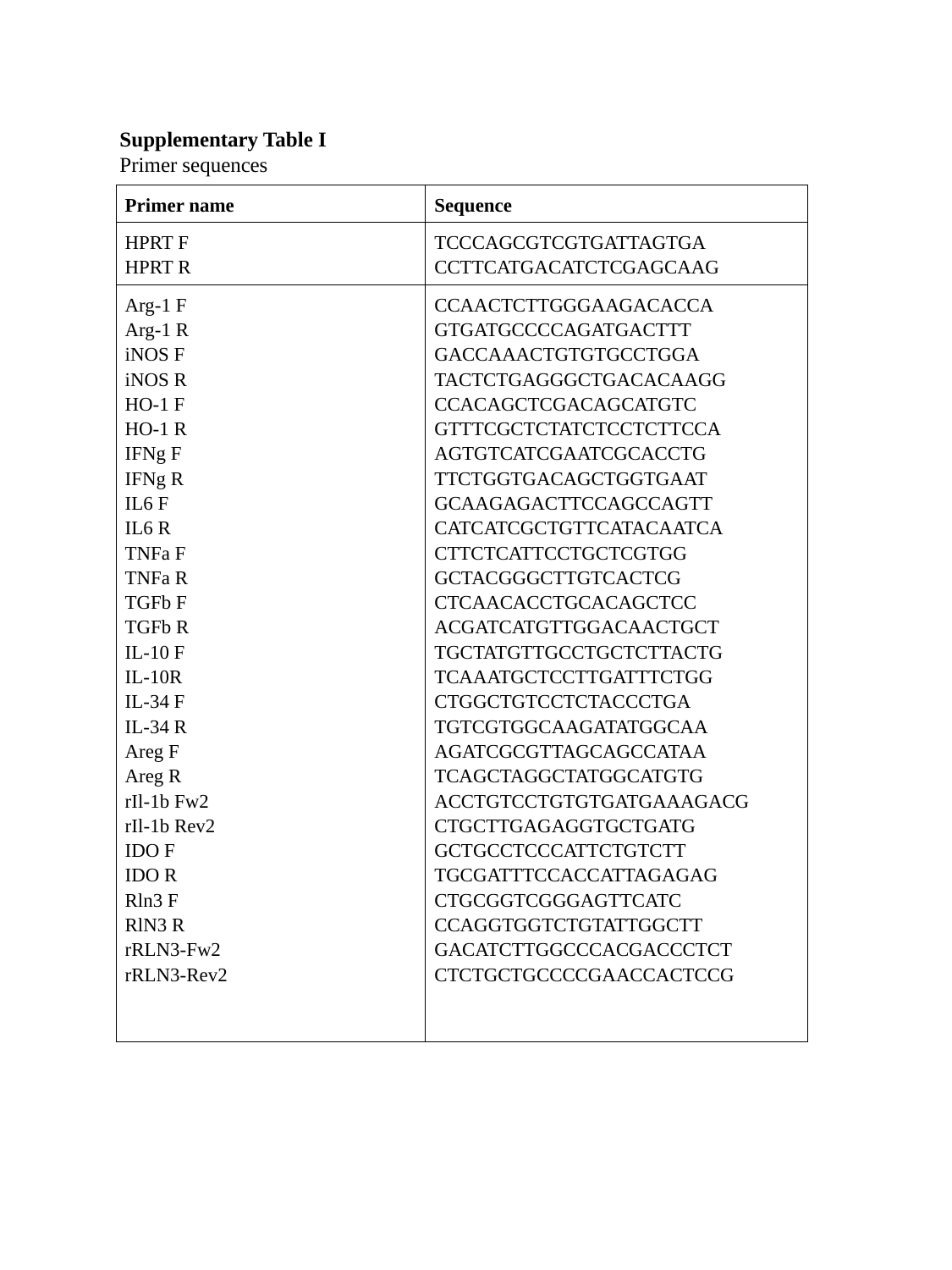

Supplementary Table I
Primer sequences
| Primer name | Sequence |
| --- | --- |
| HPRT F HPRT R | TCCCAGCGTCGTGATTAGTGA CCTTCATGACATCTCGAGCAAG |
| Arg-1 F Arg-1 R iNOS F iNOS R HO-1 F HO-1 R IFNg F IFNg R IL6 F IL6 R TNFa F TNFa R TGFb F TGFb R IL-10 F IL-10R IL-34 F IL-34 R Areg F Areg R rIl-1b Fw2 rIl-1b Rev2 IDO F IDO R Rln3 F RlN3 R rRLN3-Fw2 rRLN3-Rev2 | CCAACTCTTGGGAAGACACCA GTGATGCCCCAGATGACTTT GACCAAACTGTGTGCCTGGA TACTCTGAGGGCTGACACAAGG CCACAGCTCGACAGCATGTC GTTTCGCTCTATCTCCTCTTCCA AGTGTCATCGAATCGCACCTG TTCTGGTGACAGCTGGTGAAT GCAAGAGACTTCCAGCCAGTT CATCATCGCTGTTCATACAATCA CTTCTCATTCCTGCTCGTGG GCTACGGGCTTGTCACTCG CTCAACACCTGCACAGCTCC ACGATCATGTTGGACAACTGCT TGCTATGTTGCCTGCTCTTACTG TCAAATGCTCCTTGATTTCTGG CTGGCTGTCCTCTACCCTGA TGTCGTGGCAAGATATGGCAA AGATCGCGTTAGCAGCCATAA TCAGCTAGGCTATGGCATGTG ACCTGTCCTGTGTGATGAAAGACG CTGCTTGAGAGGTGCTGATG GCTGCCTCCCATTCTGTCTT TGCGATTTCCACCATTAGAGAG CTGCGGTCGGGAGTTCATC CCAGGTGGTCTGTATTGGCTT GACATCTTGGCCCACGACCCTCT CTCTGCTGCCCCGAACCACTCCG |
